# Galectin-3 binding protein stimulated IL-6 expression is impeded by antibody intervention in SARS-CoV-2 susceptible cell lines

**DOI:** 10.1038/s41598-022-20852-x

**Published:** 2022-10-11

**Authors:** Ana Mendes-Frias, Valentina Gallo, Valentina Iacobelli, Roberta Gentile, Giovanni Antonini, Ricardo Silvestre, Stefano Iacobelli

**Affiliations:** 1 Life and Health Sciences Research Institute (ICVS), School of Medicine, University of Minho, 4710-057 Braga, Portugal; 2 ICVS/3B’s - PT Government Associate Laboratory, Braga, Guimarães, Portugal; 3 Department of Sciences, Roma Tre University, Rome, Italy; 4 Biostructures and Biosystems National Institute (INBB), Rome, Italy; 5 MediaPharma S.r.l., Via Colonnetta, 50/A, 66100 Chieti, Italy

**Keywords:** Biochemistry, Cell biology, Drug discovery, Immunology, Diseases, Medical research

## Abstract

COVID-19 is the global pandemic that affected our population in the past 2 years. Considerable research has been done to better understand the pathophysiology of this disease and to identify new therapeutic targets, especially for severe cases. Galectin-3 (Gal-3) is a receptor present at the surface of different cell types, namely epithelial and inflammatory cells, which has been described as a severity marker in COVID-19. The activation of Gal-3 through its binding protein (Gal-3BP) is directly linked to the production of pro-inflammatory cytokines that contribute for the cytokine storm (CS) observed in severe COVID-19 patients. Here, we show that D2, a recombinant fragment of the lectin-binding region of Gal-3BP was able to stimulate the expression of IL-6 in colon and lung epithelial cell lines in β-galactoside dependent manner. We further show that D2-induced IL-6 augmentation was reduced by the anti-Gal-3BP monoclonal antibody 1959. Our data confirm and extend prior findings of Gal-3BP mediated IL-6 induction, enlightening the potential of its antibody-mediated s blockage for the prevention and treatment of CS and severe disease in COVID-19 patients.

## Introduction

Severe acute respiratory syndrome coronavirus 2 (SARS-CoV-2) is the strain of coronavirus responsible for the novel coronavirus disease 2019 (COVID-19), which is the respiratory illness responsible for the global COVID-19 pandemic. By September 13, 2022, nearly 614 million infections and over 6.5 million deaths have been reported globally^1^. COVID-19 clinical course ranges from asymptomatic cases to very severe disease and septic status which may ultimately lead to multiple organ failure^2^. Evidence accumulated so far showed that a complex interplay involving several components of the immune system, including immune-active infiltrating cells and soluble factors such as cytokines and chemokines, is mandatory for the onset of an acute severe systemic hyperinflammatory response known as 'cytokine storm' (CS), which is associated with serious adverse outcomes in patients infected with SARS-CoV-2^3^.

By the beginning of COVID-19 pandemics, numerous efforts have been made to limit virus spread and reduce mortality. Multidisciplinary approaches were often used, including the application of mathematical models^4^. Also, discovery and development of effective therapies as well as prevention strategies have benefited greatly from the application of nanotechnologies (e.g., nanoparticles, nanozymes). The use of these molecules has contributed to reduce side effects and to ameliorate delivery, circulation time and stability of antiviral drugs, immunomodulatory drugs and especially vaccines^5^. Further, some nanomaterials have been successfully used as antiviral or immunomodulatory drugs^6^. Wang et al.^7^ demonstrated an efficient anti-SARS-CoV-2 pseudovirus activity of the single-atom nanozyme containing atomically dispersed Ag atoms (Ag-TiO_2_ SAN). Studies showed the efficacy of nanomaterial-based photothermal therapy in eliciting adaptive and innate immune response. Further, studies performed on mouse models of lung cancer demonstrated the efficacy of black phosphorus (BP)-based photothermal therapy in activating adaptive and innate immunities, which could be used as therapeutical approach to combat SARS-CoV-2^8–10^. Altogether, these therapeutical strategies played a key role in preventing and treating COVID-19, drastically reducing the number of patients which progress to the severe form of the disease. Despite this, there is still no effective approach for prevention or treatment of acute respiratory distress syndrome (ARDS), that is the most common immunopathological complication of severe COVID-19 and can lead to serious tissue damage, including lung fibrosis^11^. This is mostly due to a still poor comprehension of the molecular mechanisms behind the complexity of ARDS pathogenesis. The massive production of pro- and anti-inflammatory cytokines and chemokines, including IFN-α, IFN-β, INF-γ, IL-1β, IL-6, IL-12, IL-18, IL-3, IL-10, TNF-α, TGF-β, CCL2, CCL3, CXCL8, CXCL9, and CXCL10 (IP-10) has been demonstrated to be responsible for this complication. Particularly, exaggerated production of IL-6, through induction of pro-inflammatory chemokines and cytokines, is the pioneer of the hyperinflammatory condition and CS in severe COVID-19^12^.

The cellular glycoprotein Galectin-3-binding protein (Gal-3BP, Uniprot ID—Q08380), also known as 90 K, Mac-2 BP is a multidomain, multi-functional secreted protein that has been originally identified and characterized by our group^13^ and others^14^ as a tumor associated antigen and is involved in neoplastic transformation and cancer progression^15,16^. Also, upregulation of Gal-3BP has been documented in infectious diseases, including Human Immunodeficiency Virus^17,18^ and Human Hepatitis C virus infections^19^ and recently SARS-CoV-2^20^. Functionally, Gal-3BP induces the expression and secretion of pro-inflammatory cytokines, including IL-6 in multiple different cell types, through carbohydrate-mediated interaction with Galectin-3 (Gal-3) at the cell surface^21,22^. Therefore, Gal-3BP could represent a target for modulation of CS and treatment of severe COVID-19 patients.

Here, we show that a recombinant fragment of the lectin-binding region of Gal-3BP stimulates the expression of IL-6 in SARS-CoV-2 sensitive cells, which is reduced by anti-Gal-3BP monoclonal antibody.

## Materials and methods

### Expression and purification of the recombinant Gal-3BP fragment D2

The mature Gal-3BP consists of 585 amino acids (Uniprot ID—Q08380)^23^. After cleavage of the first 18 amino acids (signal peptide), four putative regions could be distinguished in the cDNA sequence: (i) a scavenger receptor cysteine-rich domain (D1); (ii) a BTB/POZ (Broad-Complex, Tramtrack and Bric a brac/Poxvirus and Zinc finger) domain; (iii) a BACK (BTB and C-terminal Kelch) domain and (iv) an approximately 200 amino acid C-terminus with no significant similarity to other human proteins^24^. For convenience, we have named the 28 kDa DNA fragment corresponding to amino acid residues 134–288, encompassing BTP and about the first half of the BACK regions as domain 2 (D2).

MNFGLRLIFLVLTLKGVQCTRSTHTLDLSRELSEALGQIFDSQRGCDLSISVNVQGEDALGFCGHTVILTANLEAQALWKEPGSNVTMSVDAECVPMVRDLLRYFYSRRIDITLSSVKCFHKLASAYGARQLQGYCASLFAILAWSHPQFEKRGS

This is a highly glycosylated mucin-like region which binds Gal-3^24^. D2 was generated by the polymerase chain reaction (PCR). The fragment was subcloned into an Evitria’s proprietary vector system (EVITRIA, Schlieren, Switzerland) allowing the fusion in-frame to the 6xHis tag sequence that was located at the C-terminus of the domain.

D2 was transiently expressed by transfection into Chinese Hamster Ovary (CHO) cells. Concentrated culture supernatant was applied to a column of Ni-NTA Superflow (Qiagen) and D2 was eluted with increasing concentrations of imidazole according to the manufacturer’s instructions.

### Anti-Gal-3BP antibodies

Antibody 1959, a humanized variant of the murine monoclonal antibody SP-2 recognizing the human Gal-3BP was generated by identifying murine complementary determinant regions (CDRs) that were grafted onto a human antibody framework (Patent Reference WO2019197651A1). The antibody was purified on a Protein-G column (Biovision) and dialyzed against PBS.

### Cell culture and treatment

Caco-2 (epithelial cell line from human colorectal adenocarcinoma), HCC827 and A549 (epithelial cell lines from human lung adenocarcinoma) were purchased to ATCC and cultured using Dulbecco's Modified Eagle's medium supplemented with 10% Fetal Bovine Serum (Gibco), 2 mM Glutamine, 100U/mL penicillin plus 100 μg/mL streptomycin mixture and 20 mM HEPES buffer (Thermo Fisher). Cells were seeded at a concentration of 1 × 10^5^ cells/mL in a 24-well plate and incubated overnight to adhere. After adhesion, different combinations of D2 (5, 10 and 20 μg/mL), 1959 antibody (40 μg/mL), and D-lactose (50 mM) were added. After 24 h and 48 h, culture medium was collected for cytokine quantification. Cells were collected 24 h after treatment for RNA extraction.

### Galectin-3 expression

The human epithelial cell lines Caco-2, HCC827 and A549 were detached and stained with anti-human Gal-3 (Clone eBioM3/38, eBioscience, USA). Samples were acquired on LSRII flow cytometer (BD Biosciences) using the DIVA Software (Version 6.1.3) and data were analyzed using the FlowJo V10 software (Version 10.8.1).

### RNA isolation and quantitative real-time PCR (qPCR)

Total RNA was isolated using TripleXtractor (Grisp) according to manufacturer’s instructions. RNA was quantified using NanoDrop (Thermo Scientific) with ND-1000 software. RNA was converted to cDNA using Xpert cDNA Synthesis Mastermix kit (Grisp) according to manufacturer’s instructions. qRT-PCR was performed using NZYSpeedy qPCR Green Master Mix (NZYTech) in an Applied Biosystems 7500 Fast qPCR system (Applied Biosystems, Thermo Fisher Scientific). The specific primers used for IL-6 were AGTGAGGAACAAGCCAGAGC (forward) and GGTCAGGGGTGGTTATTGCA (reverse) and for 18 s were GTAACCCGTTGAACCCCATT (forward) and CCATCCAATCGGTAGTAGCG (reverse). IL-6 expression levels were normalized to 18S expression and relative expression was determined as described in Gaifem et al^25^.

### IL-6 and IP-10 measurements

IL-6 was quantified using an ELISA kit (reference 430504, BioLegend, CA, USA), according to the manufacturer's instructions. IP-10 quantification was performed with Human Macrophage/Microglia Panel Legendplex kit (ref. 740502, BioLegend), according to manufacturer’s instructions.

### Statistical analysis

Data are expressed as mean ± SD. Shapiro–Wilk test was applied to our data to evaluate normality of our variables. Once normality was confirmed in all variables, one-way ANOVA was used to identify statistical differences. For variables that reached global significance, Tukey multiple-comparison posttest was applied. All statistical analysis were carried out with GraphPad Prism (version 7.01). Statistically significant values are as follows: *p < 0.05; **p < 0.01, *< 0.001.

## Results and discussion

**Figure 1:**
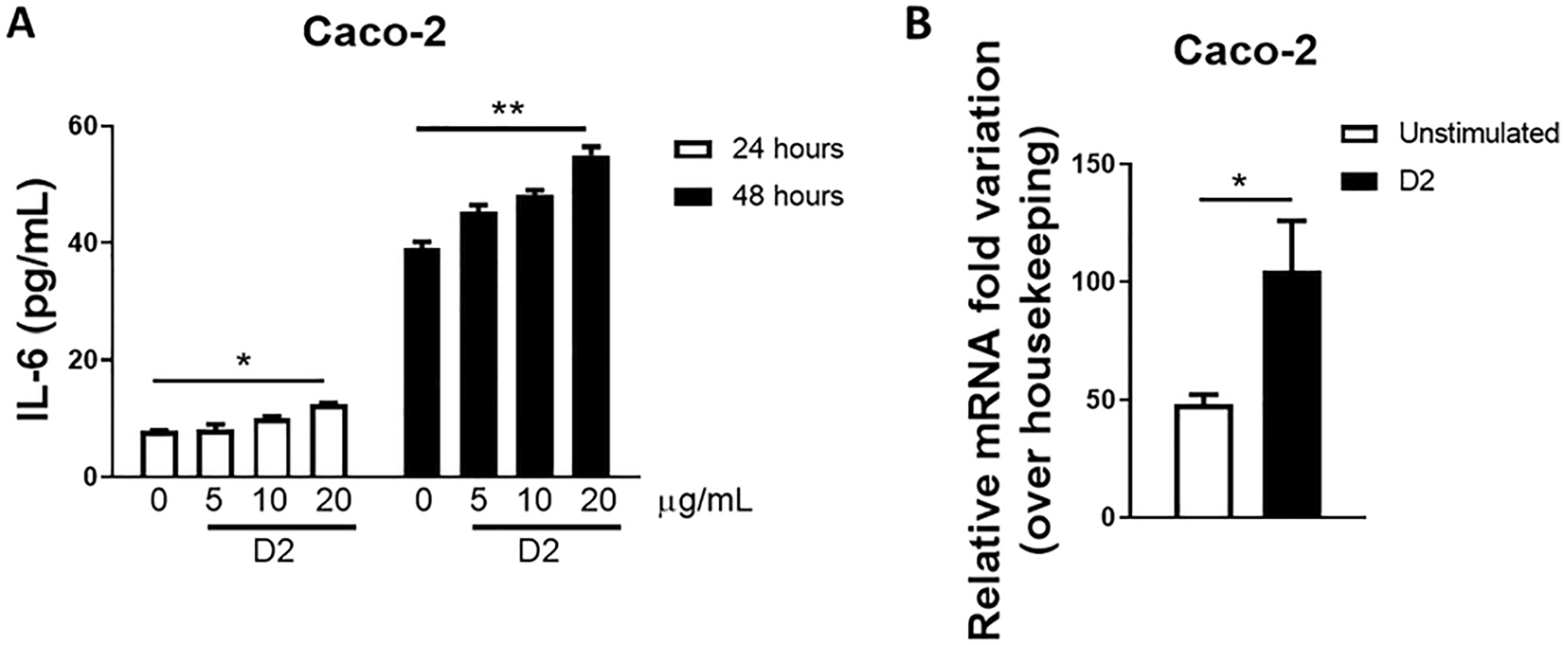
D2 Induces expression and secretion of IL6 in epithelial cells. (**A**) Caco-2 cells were incubated in the absence or presence of increasing concentrations of D2 (5–20 µg/ml) during 24 or 48 h. The secreted levels of IL-6 in culture supernatants were quantified by ELISA. (**B**) Upon 24 h of stimulation, the transcript levels of IL-6 were assessed by qPCR. The data shown are mean ± SD. *p < 0.05; **p < 0.01.

Previous reports have shown that Gal-3BP induces IL-6 expression and secretion in multiple different cell types^21,22^. The molecular mechanism involved in this process mainly resides in the interaction between Gal-3BP and Gal-3, a multifunctional β-galactoside-binding animal lectin that plays a pivotal role in modulating both chronic and acute inflammation. Indeed, the extracellular interaction of Gal-3BP with Gal-3 leads to the activation of the Ras-Mek-Erk1/2-signaling pathway, thus inducing IL-6 expression^21,22^. To evaluate whether induction of IL-6 also occurs in cells permissive to SARS-CoV-2 infection, human colon adenocarcinoma Caco-2 cells were cultured with different concentrations of D2 and the cellular supernatant was collected for IL-6 quantification. Treatment of the cells with D2 (5, 10 or 20 µg/mL) dose-dependently increased the level of IL-6 at 24 h (*p* = 0.0435) and 48 h (*p* = 0.0020) compared to untreated cells (Fig. 1A). The stimulatory effect of D2 was also observed when IL-6 expression was analysed by real-time PCR (*p* = 0.0254; Fig. 1B).

**Figure 2:**
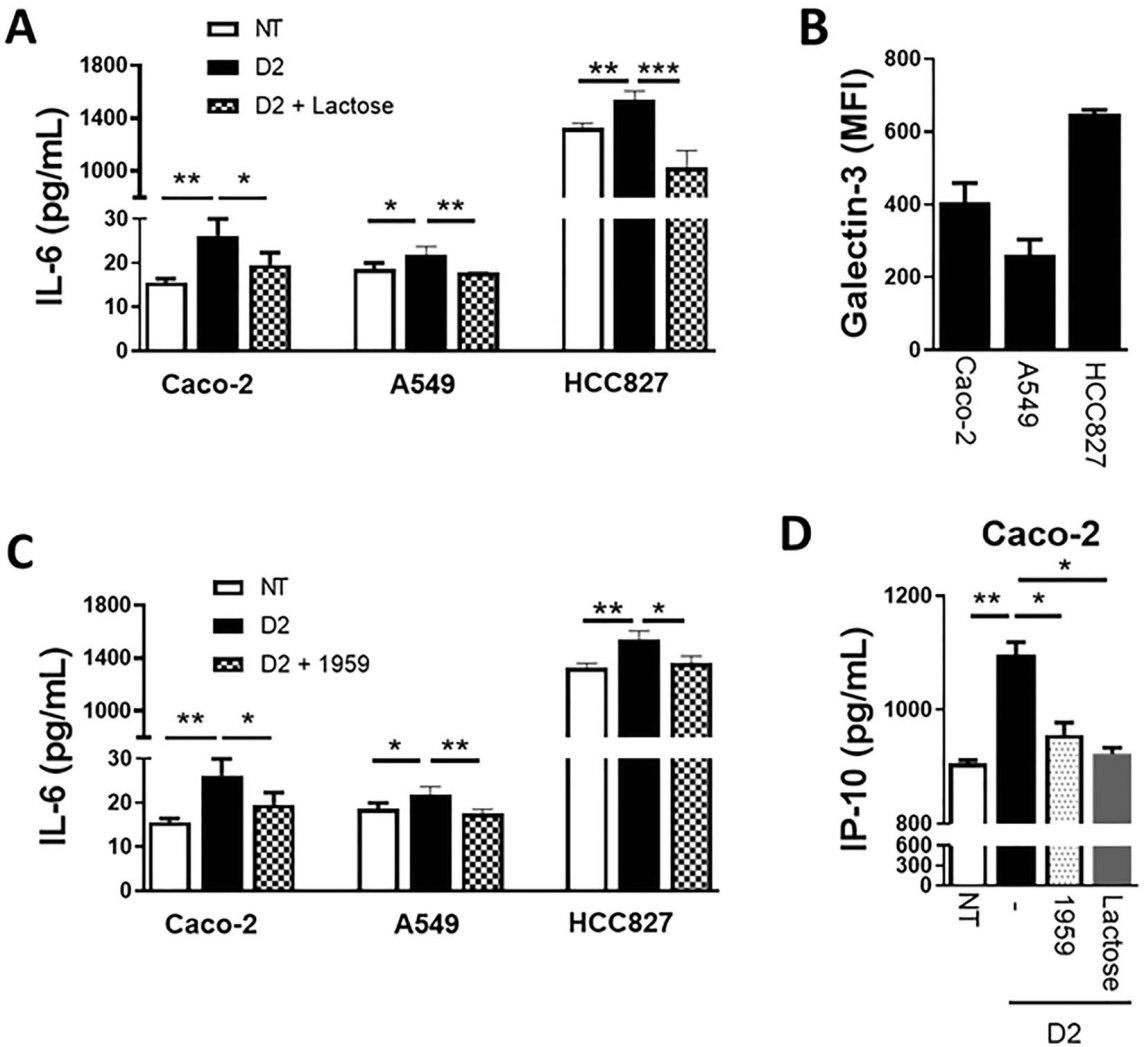
(**A**) Effect of lactose on IL-6 levels in the supernatant of Caco-2, A549 and HCC827 cells cultured without or with 20 µg/ml D2 in the presence or absence of 50 mM of lactose. The levels of IL-6 were quantified by ELISA. (**B**) Expression levels of galectin-3 on the surface of Caco-2, A549 and HCC827 cells quantified by flow cytometry. (**C**) Effect of the anti-Gal-3BP monoclonal antibody 1959 on D2-induced increase of IL-6. Cells were cultured without or with 20 µg/ml D2 in the presence or absence of 40 ug/ml of the monoclonal antibody 1959. IL-6 levels in the supernatant were measured by ELISA. (**D**) Effect of D2 alone (20 µg/ml) or in combination with 50 mM lactose or 40 ug/ml anti-Gal-3BP monoclonal antibody1959 on the level of IP-10 in culture supernatant. The data shown are mean ± SD. *p < 0.05; **p < 0.01; ***p < 0.001.

IL-6 expression induced by Gal-3BP requires β-galactoside-mediated interaction between Gal-3BP and Gal-3^26^. Then, we analyzed IL-6 secretion in the presence of lactose in Caco-2 cells and two additional SARS-CoV-2 susceptible cell lines derived from human lung adenocarcinoma, A549 and HCC827. As expected, treatment of the cells with 20 μg/ml of D2 for 48 h elicited a significant increase of IL-6 in the supernatant of all three cell lines compared to untreated controls (Fig. 2A). On the contrary, treatment with D2 in the presence of lactose (Galβ1-4glucose, 50 mM) leads to a significant decrease of IL-6 secretion compared to cells treated with D2 alone (*p* = 0.0222 for Caco-2 cells; *p* = 0.0260 for A549 cells; *p* = 0.0008 for HCC827 cells; Fig. 2A). The extent of inhibitory effect of lactose was proportional to the levels of Gal-3 expressed by the cells, being greater in HCC827 than A549 and Caco-2 cells (Fig. 2B).

Then, we evaluated whether D2 induced stimulation of IL-6 secretion could be inhibited by an anti-Gal-3BP monoclonal antibody (1959). To this end, Caco-2, HCC827 and A549 cells were pre-treated with 40 μg/ml of 1959 antibody for 20 min before adding 10 μg/ml of D2 and continuing incubation for 48 h. The augmentation of IL-6 secretion upon D2 stimulation was significantly reduced by treatment with 1959 antibody (*p* = 0.0222 for Caco-2 cells; *p* = 0.0026 for A549 cells; *p* = 0.0488 for HCC827 cells; Fig. 2C).

Finally, we evaluated the effect of D2 on IP-10 (CXCL10), as this chemokine was described to participate with IL-6 and macrophages in a network that initiates and maintains the CS during SARS-CoV2 infection^27^. An increase of IP-10 levels in the supernatant was observed after treatment of Caco-2 cells with D2, which was partially inhibited by lactose and anti-Gal-3BP monoclonal antibody (1959) (Fig. 2D).

Several studies revealed increased levels of Gal-3BP in the plasma of patients with COVID-19^20,28,29^. Consistently, these studies showed that Gal-3BP was significantly overexpressed in all symptomatic COVID-19 cohorts, with highest levels associated with severe disease. Thus, this increased production of Gal-3BP may induce a higher activation of its endogenous ligand Gal-3, promoting the release of inflammatory cytokines, which may contribute or even exacerbate pathologic proinflammatory signalling cascades in the lung and other organs^30^. Indeed, Gal-3 was recently recognized as a potential prognostic biomarker of severe COVID-19 and its inhibition has been suggested as a potential therapeutic approach to attenuate the hyperinflammation observed in these patients^31,32^. Gallo et al.^33^ recently reviewed on the anti-SARS-CoV-2 effects of Gal-3BP. Based on the collected reports, the authors raised the possibility that the Gal-3BP/galectin-3 axis could be involved in the TGF-β-mediated fibrosis observed in severe COVID-19. Other findings on the interaction between Gal-3BP and SARS-CoV-2 spike protein stimulated authors to speculate on the possibility that Gal-3BP interaction with the galectin-like NTD region of the spike protein could play an inhibitory role in virus entry^33^. These outcomes strongly suggest a potential role of Gal-3BP-Gal-3 interaction inhibitors in SARS-CoV-2’s therapeutical or prevention strategies.

In conclusion, we showed that D2, a highly glycosylated, lectin binding fragment of Gal-3BP stimulated secretion of IL-6 in SARS-CoV-2 susceptible epithelial cell lines in a Gal-3 dependent manner. Further, we showed that the stimulatory effect could be partially reverted by the anti-Gal-3BP monoclonal antibody 1959.

Overall, these findings show that targeting Gal-3BP-Gal-3 interaction with a specific antibody may represent a potential approach to reduce massive inflammation and organ damage in severe COVID-19 patients.

## Data Availability

The D2 fragment sequence is a part of the sequence of Gal-3BP_human protein deposited in Uniprot database (Uniprot ID—Q08380). The datasets used and analyzed during the current study are available from the corresponding author on reasonable request.
